# Primary pericardial mesothelioma presenting as multiple pericardial masses on CT

**DOI:** 10.1259/bjrcr.20150295

**Published:** 2016-11-12

**Authors:** Mitchell C Raeside, Kirsten Gormly, Susan J Neuhaus, Dusan Kotasek, Craig James

**Affiliations:** ^1^ Dr Jones and Partners Medical Imaging, Adelaide, SA, Australia; ^2^ Department of Surgery, University of Adelaide, Royal Adelaide Hospital, Adelaide, SA, Australia; ^3^ Adelaide Cancer Centre, Adelaide, SA, Australia; ^4^ Adelaide Pathology Partners, Adelaide, SA, Australia

## Abstract

We present the case of a 67-year-old male who was found to have multiple enhancing pericardial masses on CT imaging for investigation of weight loss and was subsequently diagnosed with primary pericardial mesothelioma. Although rare, pericardial mesothelioma is the most common primary malignancy of the pericardium and should be considered in the differential diagnosis of pericardial effusion, pericardial thickening or discreet pericardial mass. It is important for radiologists to be aware of pericardial mesothelioma as its clinical presentation is non-specific and it may be incidentally noted on radiological studies for investigation of apparently non-related symptoms. The prognosis of primary pericardial mesothelioma is universally poor.

## Summary

We present the case of a 67-year-old male who was found to have multiple enhancing pericardial masses on CT scan for investigation of weight loss and was subsequently diagnosed with primary pericardial mesothelioma. Although rare, pericardial mesothelioma is the most common primary malignancy of the pericardium and should be considered in the differential diagnosis of pericardial effusion, pericardial thickening or discreet pericardial mass. It is important for radiologists to be aware of pericardial mesothelioma as its clinical presentation is non-specific and it may be incidentally noted on radiological studies for investigation of apparently non-related symptoms. The prognosis of primary pericardial mesothelioma is universally poor.

## Clinical presentation

A 67-year-old male was initially referred for CT scan of the chest, abdomen and pelvis for investigation of a systemic illness comprising anorexia, nocturnal night sweats and approximately 10 kg weight loss. He was previously in good health and lived independently with his family. No history of significant occupational exposure (*e.g*. asbestos) was present, but he may have been exposed to asbestos in farm outbuildings during his work as a farmer.

The patient had a long-standing history of atrial fibrillation and atrial flutter. Approximately 12 months prior to presentation, he developed symptoms of pericarditis and was found to have a pericardial effusion on echocardiogram without definite solid components. He was treated clinically at that time and no pericardiocentesis was performed. Therefore, it was not possible retrospectively to determine whether there was any evidence of malignancy at the time of the echocardiogram.

## Investigations/imaging findings

**Figure 1.**
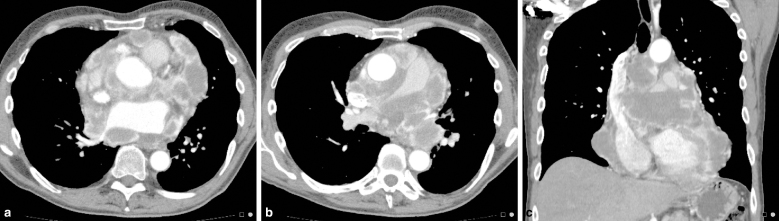
Contrast-enhanced CT scan of chest demonstrating multiple rim-enhancing, necrotic pericardial masses throughout the pericardium. (**a**) At the level of the left atrium; (**b**) mass effect on the pericardial structures with narrowing of the main pulmonary arteries; (**c**) coronal reconstruction demonstrating pericardial masses extending from the aortic arch superiorly to the diaphragm inferiorly.

Cross-sectional CT scan demonstrated extensive pericardial abnormalities. Multiple discreet rim-enhancing necrotic masses extended along the course of the pericardium from just below the level of the aortic arch to the level of the diaphragm. Mass effect was evident, with resultant narrowing of the right main pulmonary artery and compression of the pulmonary veins (Figure 1). No pericardial effusion was identified. Within the limitations of the CT scan, the myocardium appeared unremarkable. Mild fibrotic changes at the bases were evident on lung windows but there was no parenchymal lesion. No pleural plaque, calcification or mass was identified. No evidence of malignancy or metastatic disease was evident elsewhere in the chest, abdomen or pelvis.

Biochemistry analysis revealed an elevated calcium level at 3.6 mmol l^–1^ (normal range 2.2–2.6 mmol l^–1^), consistent with the diagnosis of malignant hypercalcaemia. The parathyroid hormone related peptide (PTHrP) was elevated at 6.6 pmol l^–1^ (normal range <2 pmol l^–1^). The remainder of the biochemical and haematological analysis was unremarkable.

**Figure 2.**
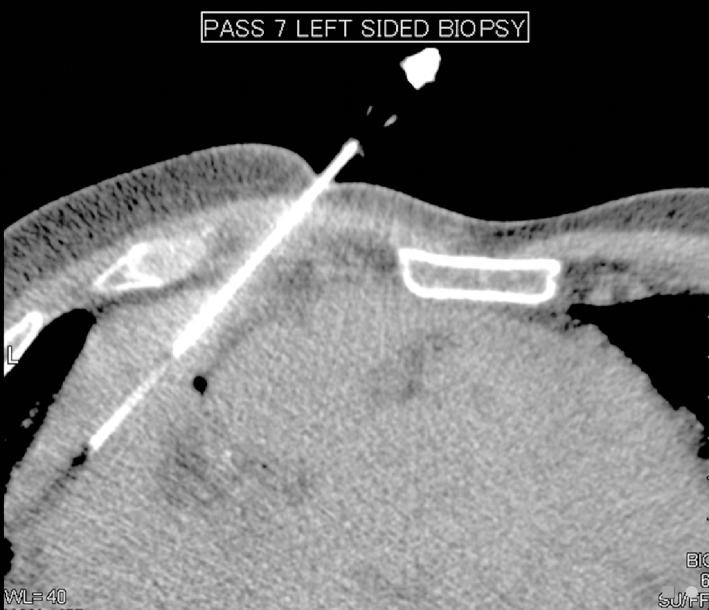
Image demonstrating core biopsy of one of the pericardial masses adjacent to the heart.

**Figure 3.**
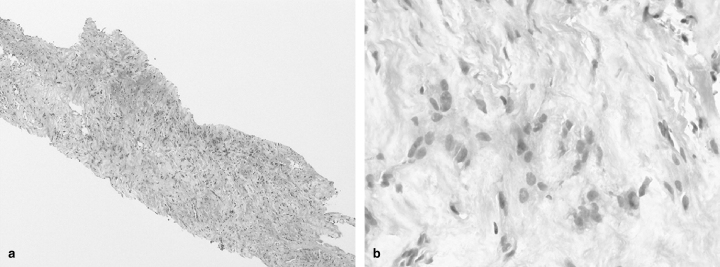
(**a**) Low power view showing spindle cell proliferation of generally low but varying cellularity and associated stromal desmoplasia (haematoxylin and eosin ×100). (**b**) High power image highlighting areas of loosely clustered, mildly atypical mesothelial cells in a fibrotic background (haematoxylin and eosin ×400).

CT-guided core biopsies of the pericardial mass (Figure 2) revealed a hypocellular, predominantly spindle cell lesion with small epithelioid foci and short storiform architecture (Figure 3). There was prominent desmoplastic stroma and focal tissue infiltration was apparent. Cytologic atypia was low grade. No necrosis, overt sarcomatous foci or zonation were noted. Immunocytochemistry demonstrated labelling of tumour cells with cytokeratin (CK) AE1/AE3 and other mesothelial markers.

Although hypocellularity and low-grade atypia with subtle tissue infiltration made diagnosis difficult, the immunocytochemistry findings and clinicoradiological correlation support the diagnosis of desmoplastic mesothelioma and argue against solitary fibrous tumour, epithelioid haemangioendothelioma and reactive perilesional fibrosis.

## Differential diagnosis

From the radiologist’s perspective, this case is a good demonstration of the differential diagnosis of pericardial masses on cross-sectional imaging. Differentials include primary and secondary tumours and non-neoplastic lesions. The most common benign pericardial lesion is a pericardial cyst. Benign tumours include fibroma, haemangioma and lipoma. The most common primary malignant tumour of the pericardium is primary pericardial mesothelioma, accounting for approximately 50% of primary pericardial malignancies. Other primary malignancies include various subtypes of pericardial sarcoma and primary pericardial lymphoma (usually diffuse large B-cell type). Germ cell tumours can also develop in the pericardium. Metastases to the pericardium are much more common than primary malignancies and are usually secondary to malignancies of the breast or lung, or melanoma. Other invasive tumours may directly involve the pericardium, such as thymic carcinoma, mediastinal teratoma or chest wall tumours. Non-neoplastic processes that can present as pericardial masses include inflammatory pseudotumour (IgG4-related disease) and tuberculous pericarditis.

## Treatment

The patient was initially treated with an infusion of pamidronate to correct his malignant hypercalcaemia. He was treated with three cycles of chemotherapy (pemetrexed and carboplatin). The lesions were deemed to be non-resectable.

## Outcome and follow-up

An interval CT was performed 3 months after the initial diagnosis for reassessment following three cycles of chemotherapy. Overall, there was a slight increase in the infiltrative pericardial malignancy, and interval development of bilateral pleural effusions. By this time, the patient had developed worsening symptoms of cardiac failure, worsening malignant hypercalcaemia and mild anaemia. His pain was relatively well controlled. The patient elected to discontinue chemotherapy and was referred to palliative care. The patient died approximately 6 weeks later, 4 months from the time of initial diagnosis.

## Discussion

Mesothelioma is a tumour arising from the mesothelial cells that line the pleural, peritoneal or pericardial surfaces, and the tunica vaginalis. Primary pericardial mesothelioma is rare, accounting for 0.7% of cases of malignant mesothelioma (with 88% arising from the pleura)^1^ and with an overall prevalence of 0.0022% in autopsy studies.^2^ Metastatic disease to the pericardium is 20–40 times more common, but primary pericardial mesothelioma is the third most common primary malignant tumour of the heart and pericardium, and the most common primary malignancy of the pericardium,^1^ It has a male predominance of approximately 2 : 1 and is most prevalent in the fourth to seventh decade of life.^3^

Unlike pleural mesothelioma, the relationship between pericardial mesothelioma and asbestos exposure is controversial, with case reports and series reporting a known asbestos association in a small number of cases.^4^

The clinical presentation is non-specific, which contributes to diagnostic challenges. Patients may present with cough, dyspnoea, orthopnoea, chest pain or constitutional symptoms such as fever, night sweats, weight loss and weakness.^4^ The clinical findings may include constrictive pericarditis, pericardial effusion, cardiac tamponade or congestive heart failure. Cardiac tamponade is a recognized complication, but is rarely the initial presentation.^3^ Pericardial mesothelioma may be found incidentally during pericardiostomy to drain a pericardial effusion or during other open heart surgery.^5^ The most common causes of death are cardiac tamponade, vena cava occlusion and congestive heart failure.^4^

There are three recognized subtypes of primary pericardial mesothelioma: epithelial, fibrous or sarcomatoid, and mixed.^2^ The epithelial variant is reported to have a more favourable prognosis, while the sarcomatoid variant is more aggressive with a poor prognosis.^6^ Tumours can occur in diffuse, multiple or localized forms, but only rarely is a dominant mass found.^1^ Bulky nodules may occur in association with both the parietal and visceral pericardium, but there is a predisposition for the diaphragmatic and pleural surfaces and extension around the major vessels.^1^ Spread to locoregional lymph nodes is common^1^ and metastases may occur, most commonly involving the lungs, kidneys and/or liver.

CT scan, MRI or echocardiography can be used to evaluate the heart and pericardium, but CT or positron emitted tomography/CT scan provides better assessment of the extent of disease. Pericardial tumours may manifest on CT/MRI as haemorrhagic effusions, pericardial thickening, enhancing nodules or pericardial masses.^7^ The role of the radiologist in the work-up of pericardial neoplasms includes describing the location, size and extent of the disease; invasion of the adjacent structures; involvement of the coronary arteries or compression of the cardiac chambers; the presence and extent of pericardial effusion or constrictive features; and identifying locoregional or distant metastatic disease.^2^ Echocardiography or CT scan can be used to guide pericardiocentesis or core biopsy, respectively.^4^

The diagnosis of pericardial sarcomatoid or desmoplastic mesothelioma can only rarely be established with cytological analysis from pericardiocentesis, given the relatively low cellularity of the fibrous or sarcomatoid variants. Similarly, core biopsy samples may not definitively establish the diagnosis and an open pericardial biopsy may be required.^6^

Desmoplastic malignant mesothelioma is a variant of the sarcomatoid subtype, characterized by paucicellular hyalinized collagen among which spindle or stellate tumour cells occur in a storiform patternless arrangement. Sarcomatoid foci are usually present^6^ and areas of necrosis and distinct tissue infiltration are required for definitive diagnosis. Only a few cases have been reported in the literature.^5,6^

Immunohistochemical analysis can be helpful, with positive mesothelial markers such as calretinin, CK5/6 and D2-40 supporting a diagnosis of mesothelioma.^8^ The diagnosis of sarcomatoid mesothelioma is more difficult, as the sensitivity and specificity of these mesothelial markers are considerably lower than for the epithelial subtype. CK AE1/AE3 is a helpful marker and has been found to show staining in cases of sarcomatoid mesothelioma.^8^

Paraneoplastic syndromes are well recognized in pleural mesothelioma but have been less well described in cases of pericardial mesothelioma, presumably owing to the relative rarity of this condition. Recognized syndromes and manifestations include thrombocytosis, syndrome of inappropriate antidiuretic hormone release, hypoglycaemia and hypercalcaemia.^9^ PTHrP has been identified in mesothelioma cells, as well as in normal and reactive mesothelial cells. Vitamin D-mediated hypercalcaemia is a recognized manifestation of pleural mesothelioma.^10^ Symptoms of hypercalcaemia may account for the initial presentation, and progression of the paraneoplastic syndrome may contribute to the patient’s clinical deterioration or death.

The prognosis for pericardial mesothelioma is universally dismal, with reported survival of between 6 weeks and 15 months regardless of treatment.^2^ Most cases are not amenable to surgical resection, and no significant benefit has been reported with radiotherapy. Chemotherapy with cisplatin/carboplatin and pemetrexed has been reported in some cases to confer a survival benefit and delay progression^3,5,6^ but most patients are ultimately treated with palliative intent.

Owing to the non-specific clinical presentation of patients with pericardial mesothelioma and the very poor prognosis associated with the disease, it is important for radiologists to be aware of this condition and consider it in the differential diagnosis of pericardial effusion, pericardial thickening or discreet pericardial masses. Diagnosis of the disease at an earlier stage may potentially allow for more definitive treatment and improve patient survival or quality of life.

## Learning points

1. Radiologists should be aware of the differential diagnosis of pericardial effusion and pericardial masses.
2. Pericardial mesothelioma is a rare but important condition to recognize, being the most common primary malignancy of the pericardium.
3. Suggesting the diagnosis of pericardial mesothelioma may lead to earlier diagnosis and treatment and allow for improved survival or quality of life.

## References

[1] Luk A, Ahn E, Vaideeswar P, Butany JW Pericardial tumors. Semin Diagn Pathol 2008; 25: 47–53. 18350922 10.1053/j.semdp.2007.12.001

[2] Restrepo CS, Vargas D, Ocazionez D, Martínez-Jiménez S, Betancourt Cuellar SL, Gutierrez FR Primary pericardial tumors. Radiographics 2013; 33: 1613–30. 24108554 10.1148/rg.336135512

[3] Patel J, Sheppard MN Primary malignant mesothelioma of the pericardium. Cardiovasc Pathol 2011; 20: 107–9. 20117025 10.1016/j.carpath.2010.01.005

[4] Godar M, Liu J, Zhang P, Xia Y, Yuan Q Primary pericardial mesothelioma: a rare entity. A rare entity. Case Rep Oncol Med 2013; 2013. 10.1155/2013/283601 PMC3697233 23840993

[5] Bang JH, Roh MS, Hong SH, Choi PJ, Woo JS Surgical experience of pericardial mesothelioma presenting as constrictive pericarditis. JC Cases 2010; 2: e96–e98. 10.1016/j.jccase.2010.04.001 PMC6265024 30524596

[6] Nicolini A, Perazzo A, Lanata S Desmoplastic malignant mesothelioma of the pericardium: description of a case and review of the literature. Lung India 2011; 28: 219–21. 21886962 10.4103/0970-2113.83985 PMC3162765

[7] Goyal N, Rojas CA, Abbara S. Pericardial disease In: Abbara S, Kalva SP, eds Problem solving in radiology: cardiovascular imaging. Philadelphia, PA: Elsevier; 2012 pp. 531–50.

[8] Marchevsky AM Application of immunohistochemistry to the diagnosis of malignant mesothelioma. Arch Pathol Lab Med 2008; 132: 397–401. 18318582 10.5858/2008-132-397-AOITTD

[9] Chahinian AP Clinical presentation and natural history of mesothelioma: pleural and pericardial In: Chahinian AP, Vogelzang NJ, Carbone M, eds Malignant mesothelioma. New York, NY; 2005 pp. 380–90.

[10] Lee JM, Pou K, Sadow PM, Chen H, Hu B, Hewison M, et al Vitamin D-mediated hypercalcemia and Cushing syndrome as manifestations of malignant pleural mesothelioma. Endocr Pract 2008; 14: 1011–16. 19095601 10.4158/EP.14.8.1011

